# Supplementary material for: Nanoparticulate Metal Oxide Top Electrode Interface Modification Improves the Thermal Stability of Inverted Perovskite Photovoltaics
Source: Nanomaterials (Basel). 2019 Nov 14;9(11):1616. doi: 10.3390/nano9111616 (PMC6915520; doi:10.3390/nano9111616)
Supplement: Supplementary file 1 [file nanomaterials-09-01616-s001.pdf]

Supplementary Materials: Nanoparticulate Metal Oxide Top Electrode Interface Modification Improves the Thermal Stability of Inverted Perovskite Photovoltaics

Ioannis T. Papadas,[1] Fedros Galatopoulos,[1] Gerasimos S. Armatas,[2] Nir Tessler,[3] and Stelios A. Choulis *,[1]

[1] Molecular Electronics and Photonics Research Unit, Department of Mechanical Engineering and Materials Science and Engineering, Cyprus University of Technology, Limassol, 3603 (Cyprus).

[2] Department of Materials Science and Technology, University of Crete, Heraklion 71003, Greece

[3] Sara and Moshe Zisapel Nano-Electronic Center, Department of Electrical Engineering, Technion-Israel Institute of Technology, Haifa 32000, Israel

*Corresponding Author: Prof. Stelios A. Choulis; E-mail: stelios.choulis@cut.ac.cy

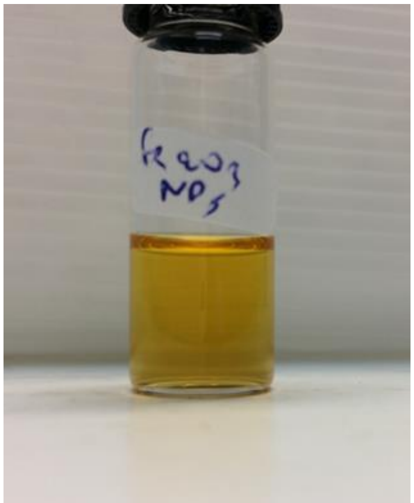

Figure S1: Dispersionof γ-Fe_2O_3 NPs in ethanol.

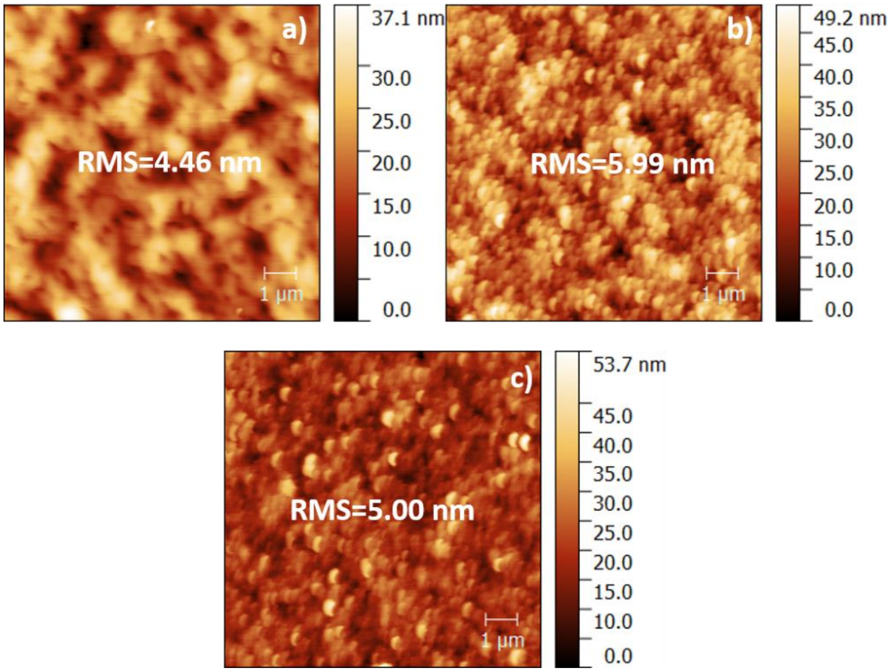

Figure S2: AFM data in 10x10 μm magnification for **a)** ITO/PEDOT:PSS/ $CH_3NH_3PbI_3$/PC[70]BM, **b)** ITO/PEDOT:PSS/ $CH_3NH_3PbI_3$/PC[70]BM/AZO and **c)** ITO/PEDOT:PSS/ $CH_3NH_3PbI_3$/PC[70]BM/γ-Fe_2O_3 films, respectively.

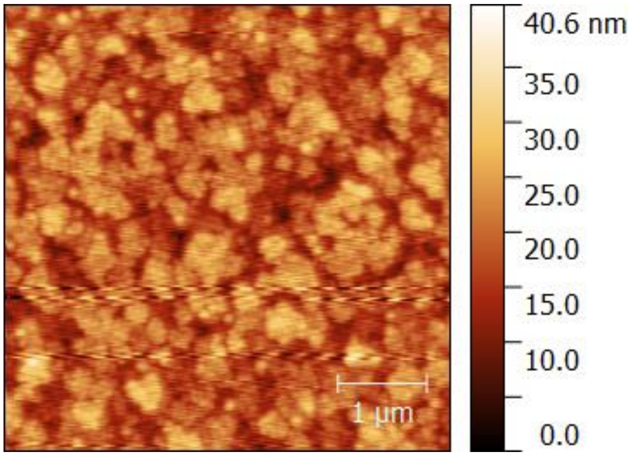

Figure S3: AMF data in 5x5 μm magnification for γ-Fe_2O_3 film on quartz substrate.

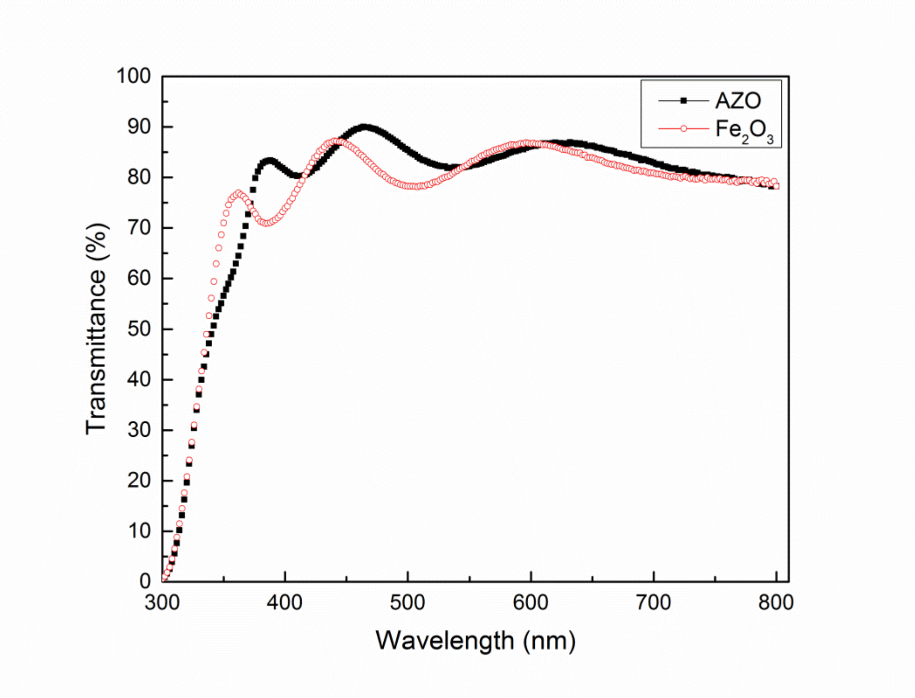

Figure S4: Transmittance spectra of AZO and γ-Fe_2O_3 films on quartz substrates
